# The effect of free-standing GaN substrate on carrier localization in ultraviolet InGaN light-emitting diodes

**DOI:** 10.1186/1556-276X-9-675

**Published:** 2014-12-13

**Authors:** Ming-Ta Tsai, Chung-Ming Chu, Che-Hsuan Huang, Yin-Hao Wu, Ching-Hsueh Chiu, Zhen-Yu Li, Po-Min Tu, Wei-I Lee, Hao-Chung Kuo

**Affiliations:** Department of Electrophysics, National Chiao Tung University, 1001 Ta Hsueh Road, Hsinchu, 30010 Taiwan; Institute of Electro-Optical Engineering, Department of Photonics, National Chiao Tung University, 1001 Ta Hsueh Road, Hsinchu, 30010 Taiwan; Advanced Optoelectronic Technology Inc., Hsinchu, 30352 Taiwan; Research Center for Applied Sciences, Academia Sinica, No. 28, Ln. 70, Sec. 2 Academia Rd, Taipei, Taiwan

**Keywords:** Ultraviolet, Light-emitting diodes, Homoepitaxially, Carrier confinement, External quantum efficiency

## Abstract

In this study, we have grown 380-nm ultraviolet light-emitting diodes (UV-LEDs) based on InGaN/AlInGaN multiple quantum well (MQW) structures on free-standing GaN (FS-GaN) substrate by atmospheric pressure metal-organic chemical vapor deposition (AP-MOCVD), and investigated the relationship between carrier localization degree and FS-GaN. The micro-Raman shift peak mapping image shows low standard deviation (STD), indicating that the UV-LED epi-wafer of low curvature and MQWs of weak quantum-confined Stark effect (QCSE) were grown. High-resolution X-ray diffraction (HRXRD) analyses demonstrated high-order satellite peaks and clear fringes between them for the UV-LEDs grown on the FS-GaN substrate, from which the interface roughness (IRN) was estimated. The temperature-dependent photoluminescence (PL) measurement confirmed that the UV-LEDs grown on the FS-GaN substrate exhibited better carrier confinement. Besides, the high-resolution transmission electron microscopy (HRTEM) and energy-dispersive spectrometer (EDS) mapping images verified that the UV-LEDs on FS-GaN have fairly uniform distribution of indium and more ordered InGaN/AlInGaN MQW structure. Clearly, the FS-GaN can not only improve the light output power but also reduce the efficiency droop phenomenon at high injection current. Based on the results mentioned above, the FS-GaN can offer UV-LEDs based on InGaN/AlInGaN MQW structures with benefits, such as high crystal quality and small carrier localization degree, compared with the UV-LEDs on sapphire.

## Background

The InGaN-based ultraviolet light-emitting diodes (UV-LEDs) with wavelength ranging from 350 to 400 nm have received enormous attention for practical application, such as bio-sensor, high density storage, and short-haul optical communication [1, 2]. Besides, the white-light LEDs (WLEDs) can be realized by using InGaN-based UV-LED chip which excited the phosphor of red, green, and blue (RGB) [3]. Compared with the case of white-light emission from yellow phosphor with blue LED chip, many researches pointed out that the InGaN-based UV-LED has many excellent properties, such as lower wavelength shift as increasing injection current, high luminous efficiency, white point determined by phosphor only, better color rendering, and stable light color. Nevertheless, there is still lack of a suitable substrate for InGaN-based UV-LED devices to develop fully their superiorities. Typically, the InGaN-based UV-LEDs were grown on sapphire substrate by hetero-epitaxial techniques, such as metal-organic chemical vapor deposition (MOCVD). However, the epitaxial layer of InGaN-based UV-LEDs still contains a high defect density (around 10^8^ to 10^10^ cm^-2^) and large strain-induced piezoelectric field due to the large lattice mismatch and the difference in thermal expansion coefficients of GaN-based films and sapphire substrates, resulting in the reduction in the internal quantum efficiency (IQE) of InGaN-based UV-LED devices. In other words, the external quantum efficiency (EQE) decreases drastically as the emission wavelength of UV-LEDs was below 400 nm. On the other hand, it was well known that the AlGaN alloy material was used as the quantum barrier (QB) of InGaN quantum wells (QWs) with low indium content to confine the electron carrier. But the two materials of AlGaN and InGaN are very different in growth temperature, which affects strongly on the quality of material and device performances. In order to overcome the problem of the above-mentioned, our group has replaced AlGaN barrier with AlInGaN quaternary material in InGaN/AlGaN MQW system, which demonstrated an improvement of 55% in output power and droop of 13% in efficiency. Besides, the AlInGaN quaternary barrier in UV-LEDs can not only improve the QB crystal quality but also increase the electron carrier concentration of QWs [4–8]. Additionally, it was believed that the optical and electrical properties of InGaN-based UV-LEDs were very sensitive to the defect density in the epilayer, suggesting that high crystalline quality epilayer could dramatically improve the device performance. The recent availability of free-standing GaN (FS-GaN) substrate with low defect density (about 10^6^/cm^2^) could facilitate this development, which can enhance the light output power, IQE, and EQE of InGaN-based UV-LEDs.

Therefore, in order to further improve the performance of UV-LED devices based on the InGaN/AlInGaN MQW structure, in the present study, we will introduce the FS-GaN substrate for the homo-epitaxial growth of high-quality InGaN-based UV-LED devices based on the InGaN/AlInGaN MQW structure. We also examine the effect of FS-GaN on the growth of UV-LEDs based on the InGaN/AlInGaN MQW structure with the characterizations of interface structure and optical properties being emphasized.

## Methods

### Fabrication of UV-LEDs

**Figure 1:**
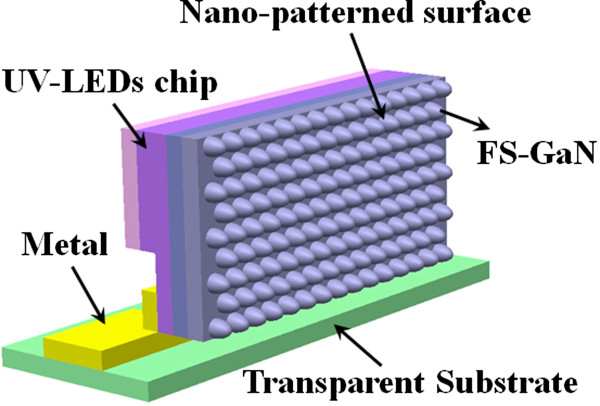
**Schematics of UV-LEDs based on InGaN/InAlGaN MQW structures on FS-GaN substrate by using VTP.**

All UV-LEDs based on the InGaN/AlInGaN MQW epitaxial structure were grown by a commercial atmospheric pressure metal-organic chemical vapor deposition (AP-MOCVD) system (model: Nippon SR4000, Taiyo-Nippon Sanso) with a horizontal reactor in the same run. The MO compounds of TMGa, TMIn, TMAl, and gaseous NH_3_ were employed as the reactant source materials for Ga, In, Al, and N, respectively, and H_2_ and N_2_ were used as the carrier gas. The substrates employed herein were 2-in. FS-GaN with 300 μm in thickness, which was fabricated by hydride vapor phase epitaxy (HVPE). The epitaxial structure of the UV-LEDs was grown on both substrates, comprising a 2.5-μm-thick n-GaN epilayer grown at 1,150°C, a 12-period InGaN/AlInGaN MQW active layer grown at 830°C, 15-nm-thick Mg-doped Al_0.3_Ga_0.7_N electron blocking layers (EBLs) grown at 1,030°C, and a 100-nm-thick p-GaN layer grown at 1,030°C. In addition, the growth of UV-LEDs based on the InGaN/AlInGaN MQW epitaxial structure on undoped GaN/sapphire template was also conducted for comparison. In order to compare both samples, we regrow the UV-LED epitaxial structure on the FS-GaN substrate firstly. We optimized the growth temperature of MQWs and then grow the same UV-LED epitaxial structure on the sapphire substrate based on the photoluminescence (PL) wavelength of the UV-LED epitaxial structure on the FS-GaN substrate. After epitaxial growth, the indium tin oxide (ITO) film (180 nm) was first deposited on the UV-LEDs as a transparent contact layer (TCL). Then, the surfaces of the UV-LEDs which were partially etched until the 1.5-μm depth of the n-GaN layers were exposed. We subsequently deposited Cr/Pt/Au (50 nm/50 nm/150 nm) onto the exposed n-GaN and p-GaN layers to serve as the n-type and p-type electrodes, respectively. After that, UV-LEDs were cut into square pieces with a dimension of 300 × 300 μm^2^. Finally, the blue LED chip was packaged by a vertical transparent package (VTP) to enhance light extraction efficiency [9]. The schematics of UV-LEDs based on InGaN/AlInGaN MQW structures on the FS-GaN substrate by using VTP is depicted in Figure 1.

The surface morphology of UV-LED epilayers was observed by atomic force microscopy (AFM) with a scanning area of 10 μm × 10 μm. The crystalline quality and interface of our epitaxial structures was evaluated by high-resolution double-crystal X-ray diffraction (HRXRD, D8) using Cu Kα radiation as the X-ray source (*λ* = 1.54056 Å). The room temperature Raman scattering was used to analyze the residual strain of GaN epitaxial layers and UV-LEDs. The interfacial microstructures and In distribution of the epilayer were observed by high-resolution transmission electron microscopy (HRTEM) and energy-dispersive spectrometer (EDS) mapping. The optical properties were investigated by temperature-dependent PL measurement. Finally, the light-current-voltage (*L*-*I*-*V*) characteristics of UV-LED devices were measured at room temperature under continuous-wave (CW) operation.

## Results and discussion

**Figure 2:**
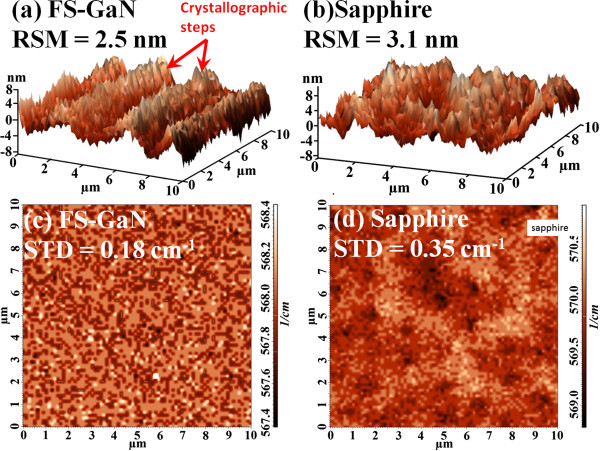
**AFM and micro-Raman images for UV-LEDs grown on (a, c) FS-GaN and (b, d) sapphire substrates.**

Figure 2 shows the AFM images for the UV-LEDs grown on FS-GaN and sapphire substrates. The root mean square (RMS) of UV-LEDs on sapphire was about 3.1 nm, as shown in Figure 2b. It can be seen that the RMS was decreased to 2.5 nm as the FS-GaN was used, as shown in Figure 2a. Besides, the uniform, well-defined, and long crystallographic steps can be observed on the top surface of UV-LEDs on FS-GaN, suggesting that the UV-LEDs based on InGaN/AlInGaN MQWs of excellent quality are grown. On the other hand, Figure 2c,d displays the micro-Raman mapping images of specimens, within a scanning area of 10 μm × 10 μm. In general, the Raman spectra show two Raman shift peaks: one can be attributed to the *E*_2_ (high) mode of the GaN epilayer and another can be attributed to the *A*_1_ (LO) mode of the GaN epilayer. Therefore, the average Raman shift peak of *E*_2_ (high) for the UV-LEDs grown on FS-GaN and sapphire substrates was located at around 567.8 and 570.4 cm^-1^, respectively. We can calculate the average strain value of specimen by Equation 1 [10]:

1

where Δ*ω* is the Raman shift peak difference between the strained GaN epilayer and the strain-free GaN epilayer and *C* is the biaxial strain co-efficient, which is 2.25 cm^-1^/GPa [11]. The calculated in-plane compressive stress was about 0.71 and 1.86 GPa for the UV-LEDs grown on FS-GaN and sapphire substrates, respectively. Compared with the UV-LEDs on sapphire, the strain relaxation degree of the UV-LEDs on FS-GaN is about 62%. Additionally, the fluctuation of Raman shift peak over the area scanned is denoted as standard deviation (STD in cm^-1^). As can been seen, the STD of FS-GaN and sapphire case is estimated to be about 0.18 and 0.35 cm^-1^, respectively. It is obvious that compared with the UV-LEDs on sapphire samples, the UV-LEDs on FS-GaN showed a quite uniform Raman peak distribution over the region scanned, where the STD for the UV-LEDs on FS-GaN is only 0.18 cm^-1^. Consequently, we can expect that the UV-LEDs grown on the FS-GaN substrate have weaker quantum-confined Stark effect (QCSE) and small curvature [12].

**Figure 3:**
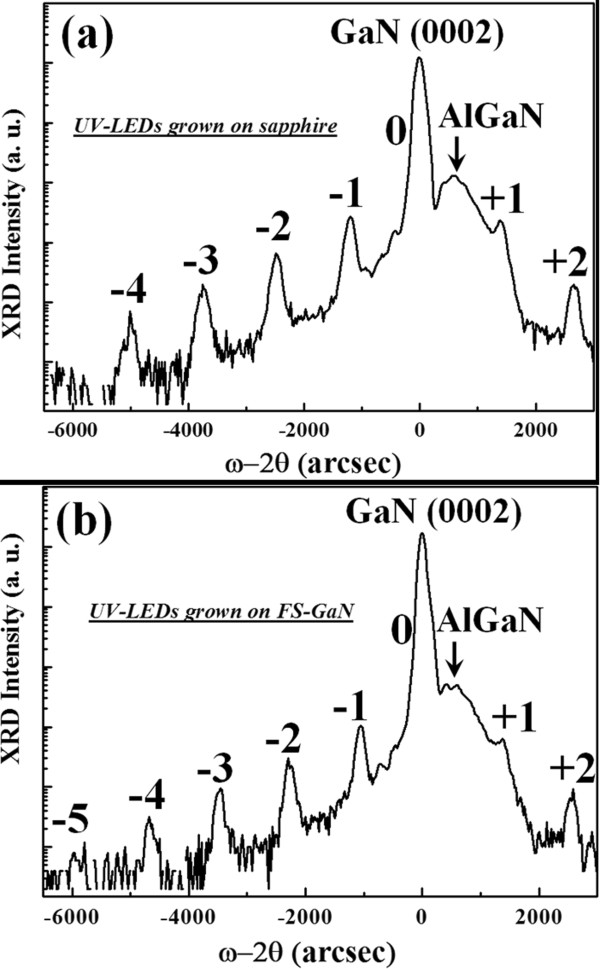
**(0002) reflection HRXRD ω/2θ curves of UV-LEDs grown on (a) sapphire and (b) FS-GaN substrates.**

Figure 3 shows the HRXRD *ω*/2*θ* scan for the InGaN/AlInGaN multi-quantum well (MQW) UV-LED epitaxial structures on (a) sapphire and (b) FS-GaN. The interface roughness (IRN) of MQW structures fabricated on these substrates was further analyzed by using Equation 2 [13, 14]:

2

where *n* is the order of the satellite, Λ and *σ*/Λ are the period of satellite peak and IRN, respectively, Δ*θ*_M_ is the angle distance between adjacent satellite peaks, and *W*_0_ and *W*_*n*_ are the full width at half maximum of the zeroth- and *n*th-order peaks, respectively. We can estimate that the IRN of the UV-LEDs grown on the FS-GaN substrate (1.6%) is smaller than that of the UV-LEDs grown on the sapphire substrate (2.7%). As indicated in ref. [13], the IRN of MQWs is affected by the defects, microstructure, and phase separation in MQWs. Therefore, our X-ray analysis results might manifest that the crystalline quality of the MQW epitaxial structure grown on the FS-GaN substrate is superior to those grown on sapphire substrates.

**Figure 4:**
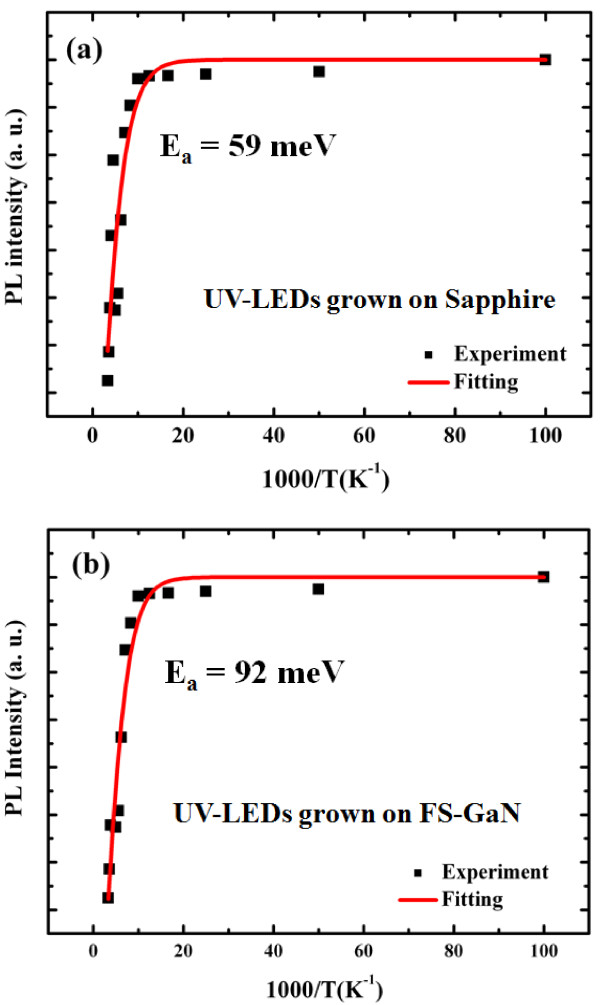
**Temperature dependence of PL intensity for UV-LEDs grown on (a) sapphire and (b) FS-GaN substrates.**

**Figure 5:**
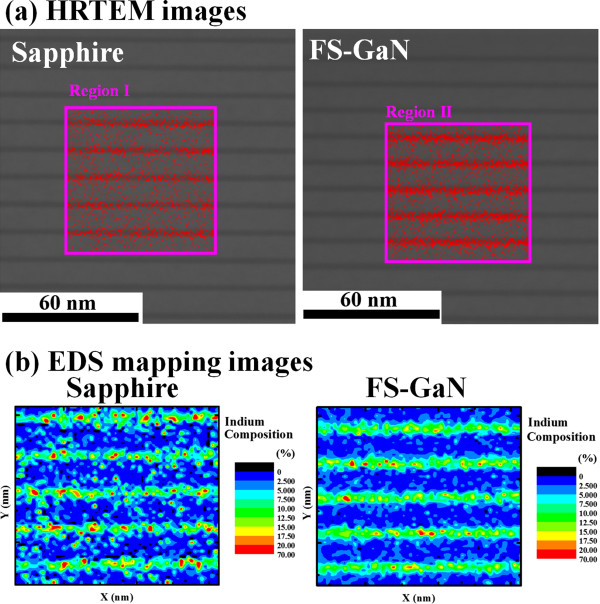
**HRTEM images (a) and EDS mapping images (b) of UV-LEDs grown on sapphire and FS**-**GaN substrates.**

Figure 4 shows the temperature dependence of integrated PL intensity for the UV-LEDs grown on (a) sapphire and (b) FS-GaN substrates. Evidently, the temperature dependence of integrated PL intensity can be well fitted by Arrhenius formula [15]. By the fitting, the activation energy, *E*_a_, was estimated to be 59 and 92 meV for UV-LEDs based on the InGaN/AlInGaN MQW structure grown on sapphire and FS-GaN substrates, respectively. Here, the *E*_a_ can be an indication of effective energy depth of QWs. Therefore, the activation energy of UV-LEDs on the FS-GaN substrate is 8.6% higher than that of the UV-LEDs on sapphire, leading to a minor overflow of carriers outside the InGaN MQW active region. That is to say, the carrier in UV-LEDs grown on the FS-GaN substrate exhibited the larger carrier confinement effect and sharp interface between QWs and QB due to high-quality MQWs.To further understand the effect of substrate on the uniformity of InGaN/AlInGaN MQWs, the HRTEM analysis of the InGaN/AlInGaN MQW region was performed, as shown in Figure 5a. Besides, the EDS mapping was used to observe more clearly the indium (In) distribution in the InGaN/AlInGaN MQW region. Hence, both the EDS mapping images of regions I and II in Figure 5a were taken and presented in Figure 5b. As shown in Figure 5, the disorder is relatively evident for the UV-LEDs on sapphire substrates. Microstructures were formed with the spacing between them of about 1 to 2 nm or intimately connected to one another without any spacing between them for the UV-LEDs on the sapphire substrate. The non-uniform In distribution and phase separation in the MQW region also can be found in the same figure. However, when the UV-LEDs were grown on the FS-GaN substrate, the MQWs exhibited very uniform, and no microstructure and phase separation were found. It is notable that these two specimens were produced completely in the same run and therefore the growth conditions for these two specimens compared were completely the same except the substrates used. Consequently, it was very clear that the homo-epitaxial growth on FS-GaN should be the main cause for the uniform distribution of indium, no phase separation of InGaN well, and no microstructures.

**Figure 6:**
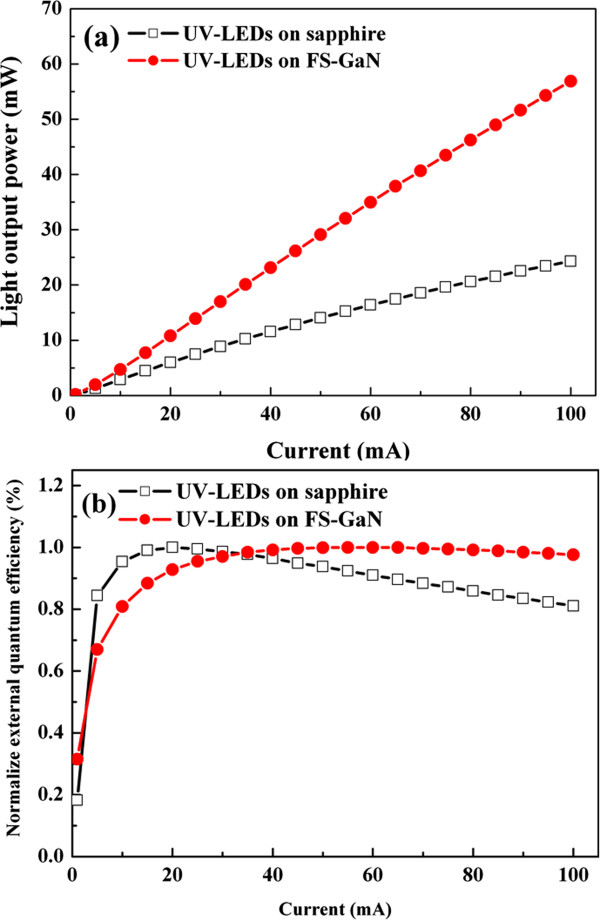
**Forward current and normalized external quantum efficiency. (a)** Forward current as a function of light output for the UV**-**LEDs grown on sapphire and FS**-**GaN substrates. **(b)** Normalized external quantum efficiency as a function of forward current for LEDs on sapphire and FS-GaN under CW mode.

Figure 6a shows the typical power-current (*L*-*I*) characteristics for both samples. As we can see, the output power of the UV-LEDs grown on the FS-GaN substrate was enhanced by 78% at 20 mA compared with the UV-LEDs grown on the sapphire substrate. Finally, Figure 6b shows the EQE as a function of forward current for the UV-LEDs grown on FS-GaN and sapphire substrates. The EQE of these two types of UV-LEDs are under CW operations. Therefore, the efficiency droop, defined as (*η*_peak_ - *η*_100 mA_)/*η*_peak_, was reduced from 20% in the UV-LEDs grown on the sapphire substrate to 3% in the UV-LEDs grown on the FS-GaN substrate. Clearly, the performance of UV-LEDs on FS-GaN can be expected due to ultra-low dislocation density and high thermal conductivity of FS-GaN. Comparing these measurements, the use of the FS-GaN substrate is not only to improve the crystal quality of UV-LED device but also to reduce the efficiency droop under high injection current.

The results demonstrated in Figures 2, 3, 4, 5, and 6 can be understood as follows. In general, the quantum-dot-like structures in traditional InGaN/AlGaN MQWs could be responsible for the surprisingly high quantum efficiencies [16–19]. Nevertheless, the non-uniform distribution of indium/aluminum and phase separation of InGaN/AlGaN will procure the formation of microstructures in MQWs [20], which induces the localization of carriers within the MQW stack, and therefore raise the IRN of MQWs [21]. However, the lowest STD exhibited by the UV-LEDs on FS-GaN might imply this sample to be with the lowest number of microstructures within its MQWs, and this is considered to be connected to a smallest IRN thus obtained. Additionally, the low threading dislocation density may be responsible for the high uniform electron and hole distribution in the MQW region of the UV-LEDs on FS-GaN. A direct consequence is the increase in the carrier distribution and radiative recombination rate in the MQW region and a rise in the light output power. Therefore, both features of uniform carrier distribution, weak QCSE, and small strain in the UV-LEDs on FS-GaN can be attributed to that a relatively good-quality epitaxial structure has been grown on the FS-GaN substrate due to homo-epitaxial growth.

## Conclusions

We investigate the influence of the FS-GaN substrate on the performance of UV-LEDs grown atop by AP-MOCVD. The micro-Raman shift peak mapping image indicated that the strain-free GaN-based epilayer is grown on the FS-GaN substrate. Additionally, the effect of the FS-GaN substrate on the quality and In distribution of MQWs in UV-LEDs was examined in detail by HRTEM and EDS mapping. HRXRD analyses demonstrated high-order satellite peaks and clear fringes between them for the UV-LEDs grown on the FS-GaN substrate, from which the IRN was estimated to be 1.6%. From the temperature-dependent PL measurement, the UV-LEDs grown on the FS-GaN substrate have better carrier confinement than those grown on the sapphire substrate. Based on the results mentioned above, the light output power of the UV-LEDs on the FS-GaN substrate is higher than that of the UV-LEDs on the sapphire substrate by 78% at 20 mA. Besides, the efficiency droop was reduced from 20% in the UV-LEDs grown on the sapphire substrate to 3% in the UV-LEDs grown on the FS-GaN substrate. Conclusively, the use of the c-plane FS-GaN substrate suggests an effective technique to fabricate thereon high-power UV-LEDs.

## Abbreviations

AFM: atomic force microscopy
AP-MOCVD: atmospheric pressure metal-organic chemical vapor deposition
EDS: energy-dispersive spectrometer
EQE: external quantum efficiency
FS-GaN: free-standing GaN
HRTEM: high-resolution transmission electron microscopy
HRXRD: high-resolution X-ray diffraction
HVPE: hydride vapor phase epitaxy
IQE: internal quantum efficiency
IRN: interface roughness
ITO: indium tin oxide
LEDs: light-emitting diodes
PL: photoluminescence
QCSE: quantum-confined Stark effect
TCL: transparent contact layer
UV: ultraviolet
VTP: vertical transparent package.
